# Expression of protein-tyrosine phosphatases in Acute Myeloid Leukemia cells: *FLT3 ITD sustains high levels of DUSP6 expression*

**DOI:** 10.1186/1478-811X-10-19

**Published:** 2012-07-11

**Authors:** Deepika Arora, Susanne Köthe, Monique van den Eijnden, Rob Hooft van Huijsduijnen, Florian Heidel, Thomas Fischer, Sebastian Scholl, Benjamin Tölle, Sylvia-Annette Böhmer, Johan Lennartsson, Fabienne Isken, Carsten Müller-Tidow, Frank-D Böhmer

**Affiliations:** 1 Institute of Molecular Cell Biology, Center for Molecular Biomedicine, Jena University Hospital, Jena, Germany; 2 Merck Serono, Geneva, 1202, Switzerland; 3 Department of Hematology/Oncology, Otto-von Guericke-University, Magdeburg, Germany; 4 Department of Hematology/Oncology, Clinic for Internal Medicine II, Jena University Hospital, Jena, Germany; 5 Ludwig Institute for Cancer Research, Uppsala Branch, Uppsala, Sweden; 6 Department of Medicine A, Hematology and Oncology, University of Münster, Münster, Germany

**Keywords:** Acute myeloid leukemia, Protein-tyrosine phosphatases, Dual-specificity phosphatases (DUSP), mRNA expression, Fms-like tyrosine kinase (FLT3) with internal tandem duplication (ITD), DUSP6, ERK signaling

## Abstract

Protein-tyrosine phosphatases (PTPs) are important regulators of cellular signaling and changes in PTP activity can contribute to cell transformation. Little is known about the role of PTPs in Acute Myeloid Leukemia (AML). The aim of this study was therefore to establish a PTP expression profile in AML cells and to explore the possible role of FLT3 ITD (Fms-like tyrosine kinase 3 with internal tandem duplication), an important oncoprotein in AML for PTP gene expression. PTP mRNA expression was analyzed in AML cells from patients and in cell lines using a RT-qPCR platform for detection of transcripts of 92 PTP genes. PTP mRNA expression was also analyzed based on a public microarray data set for AML patients. Highly expressed PTPs in AML belong to all PTP subfamilies. Very abundantly expressed PTP genes include *PTPRC, PTPN2, PTPN6, PTPN22, DUSP1, DUSP6, DUSP10, PTP4A1, PTP4A2, PTEN,* and *ACP1*. PTP expression was further correlated with the presence of FLT3 ITD, focusing on a set of highly expressed dual-specificity phosphatases (DUSPs). Elevated expression of *DUSP6* in patients harboring FLT3 ITD was detected in this analysis. The mechanism and functional role of FLT3 ITD-mediated upregulation of *DUSP6* was then explored using pharmacological inhibitors of FLT3 ITD signal transduction and si/shRNA technology in human and murine cell lines. High *DUSP6* expression was causally associated with the presence of FLT3 ITD and dependent on FLT3 ITD kinase activity and ERK signaling. DUSP6 depletion moderately increased ERK1/2 activity but attenuated FLT3 ITD-dependent cell proliferation of 32D cells. In conclusion, DUSP6 may play a contributing role to FLT3 ITD-mediated cell transformation.

## Lay abstract

In Acute Myeloid Leukemia (AML), cells in the bone marrow, which normally give rise to functioning blood cells like monocytes, have stopped their differentiation at an early immature state. Moreover, the cells divide rapidly and are largely autonomous, i.e. independent form extracellular signals, in their capacity to proliferate. The molecular reasons for these defects are only partially understood. An important oncoprotein, which drives proliferation of leukemic cells in a subset of AML patients, is named FLT3 ITD. ITD (internal tandem duplication) stands for a molecular alteration which makes this molecule, an enzyme catalyzing the transfer of phosphate from ATP to tyrosine residues of proteins (a protein-tyrosine kinase), highly and constitutively active. This leads to many alterations in the affected cells, including the re-programming of gene expression. In this study we have analyzed most members of an enzyme family designated protein-tyrosine phosphatases (PTPs, enzymes which revert the action of protein-tyrosine kinases by removing phosphate residues from phosphorylated tyrosines) for their abundance in AML cells. Highly expressed PTPs may play a contributing role for leukemic cell proliferation, lowly expressed members may be required for regulation of normal cell proliferation. We observed that among the analyzed 92 genes, one particular PTP designated DUSP6 is selectively highly expressed in such AML cells which also harbor the oncoprotein FLT3 ITD. Functional studies suggest that this PTP seems to contribute to the undesired cell proliferation driven by FLT3 ITD. It may therefore be an interesting candidate as drug target.

## Background

Protein-tyrosine phosphatases (PTP) are important regulators of cellular signal transduction [1]. Several types of alterations of specific PTP functions have been reported in cancer cells, including gene deletion, allele loss, reduced expression by promoter methylation, or inactivation by point mutation or oxidation, all leading to loss-of function [1,2]. Upregulation of expression, or gain-of-function mutations have also been found for members of the PTP family, which have been shown or presumed to promote oncogenesis [1-4].

Acute myeloid leukemia (AML) is the most frequent leukemia in adults. Treatment options for AML are still very limited [5], and the identification of suitable targets for novel therapies is highly warranted. The analysis of alterations in signal transduction in AML cells may reveal novel potential targets for therapy. Little is known about the role PTPs may play in deregulated signal transduction in AML.

Fms-like tyrosine kinase 3 (FLT3) is a class-III receptor-tyrosine kinase, which is frequently mutated in AML cells. Mutations resulting in internal tandem duplications (ITD) of sequences of different length in the juxtamembrane domain or the first part of the kinase domain of FLT3 cause strong FLT3 activation, associated with elevated and altered signal transduction. Notably, in addition to activation of ERK1/2 and AKT, FLT3 ITD can potently activate STAT5 [6,7]. We have previously characterized PTPs involved in FLT3 signaling: SHP-1/PTPN6 and PTP1B/PTPN1, but not SHP-2/PTPN11 can dephosphorylate FLT3 upon transient coexpression [8]. Consistent with findings of Heiss et al. [9], SHP-2/PTPN11 was found to mediate ERK1/2 activation and proliferation by wildtype FLT3, but appeared dispensable for transformation by FLT3 ITD [10]. Using an shRNA-based screen, we identified DEP-1/PTPRJ as a negative regulator of FLT3 autophosphorylation and signaling [11]. Very recently, PRL-3/ PTP4A3 has been identified as a downstream mediator of FLT3 signaling [12].

Gene expression analysis has been applied to AML cells to identify signatures for AML subtypes and potential predictors for prognosis and treatment [13-16]. Little has, however, been reported about PTP gene expression in AML cells. Alterations in PTP expression in AML cells may lead to silencing of PTPs with tumor-suppressing functions, or enhance abundance of pro-oncogenic PTPs. Along these lines, downregulation of mRNA expression of SHP-1/PTPN6, a potential negative regulator of FLT3 signaling, has been reported in presence of FLT3 ITD [17]. To obtain a more comprehensive picture of PTPs, which may be relevant in the context of AML, we have analyzed PTP mRNA expression in AML patient cells and cultured AML cell lines. We employed a RT-qPCR platform established for 92 members of the PTP family [18], and compared these results with data from published expression mRNA arrays. Among the relatively highly expressed PTPs were several dual-specificity PTPs (DUSPs). We correlated expression of several DUSPs with the FLT3 status, and found a robust upregulation of *DUSP6* mRNA as well as DUSP6 protein associated with FLT3 ITD expression. DUSP6 is an important negative regulator of the RAS-ERK pathway, based on its capacity to potently dephosphorylate the pThr-X-pTyr motif in ERK1/2 [19,20]. We could recapitulate a negative regulation of ERK1/2 by DUSP6 in FLT3 ITD-expressing cells. Surprisingly, reduction of DUSP6 protein by shRNA did not enhance but appeared to diminish cell proliferation in this system, indicating a contributing role for DUSP6 in sustaining FLT3 ITD-dependent cell proliferation.

## Results

### Expression of PTP genes in AML cells

We first intended to obtain an overview of PTP expression in AML cells. mRNA expression of 92 PTP genes was analyzed by RT-qPCR in primary AML cells (n = 9) and compared with expression assessed in primary AML cells by Affymetrix gene chips (n = 206) [14]. We also performed PTP expression analysis by RT-qPCR in the AML cell lines THP-1, EOL-1, MV4-11, and RS4-11 (Table 1). These cells were chosen since they represent different AML subtypes. Moreover, they are widely used to assess signaling of AML-related oncoproteins. Notably, MV4-11 cells harbor the oncogenic version of FLT3, FLT3 ITD, whereas the other cell lines express wildtype FLT3. Affymetrix and RT-qPCR analysis results were to a large extent in agreement with respect to identification of abundantly expressed PTP genes, with some exceptions discussed later. Highly expressed PTPs were found in all PTP classes. The most abundantly expressed transmembrane PTPs were *PTPRC* (common protein name CD45), and *PTPRA* (RPTPα) in all samples. Other transmembrane PTPs showed relatively low-level expression. *PTPRJ* (DEP-1, CD148), and *PTPRR* were, however, still clearly detectable by RT-qPCR in the patient samples. Only *PTPRJ* was well detectable also in all the cell lines, whereas *PTPRR* mRNA was only prominently expressed in RS4-11 cells. Among the non-receptor classical PTPs (NRPTPs), *PTPN2* (TC-PTP), *PTPN6* (SHP-1), *PTPN7* (HePTP), *PTPN11* (SHP-2), *PTPN12* (PTP-PEST), and *PTPN22* (Lyp) were expressed most abundantly. High-level expression was observed for several members of the MAPK-kinase phosphatase (MKP) family of dual-specificity phosphatases (DUSPs): *DUSP1* (MKP-1), *DUSP2* (PAC-1), and *DUSP6* (MKP-3). *DUSP1* and *DUSP10* were highly expressed in patient samples. *DUSP1* and *DUSP6* were even the two most highly expressed of all PTPs analyzed. Expression of these DUSP species was, however, much lower in the cell lines. mRNA of *DUSP7* and of a catalytically inactive DUSP, *MK-STYX*, were clearly detectable in all samples, but had relatively lower expression in the primary AML cells. Many atypical DUSPs were expressed at easily detectable mRNA levels, including *DUSP3, DUSP12, DUSP22, DUSP23, PTPMT1*, and the catalytically inactive atypical *DUSP STYX*. Of the other subfamilies, *SSH2, PTP4A1* (PRL-1), *PTP4A2* (PRL-2), *PTEN*, and the myotubularins *MTMR2MTMR4MTMR5*, and *MTMR6* were well detectable. *ACP1* (LMW-PTP) was abundantly expressed in all samples.

**Table 1:** Protein-tyrosine phosphatase (PTP) gene expression in Acute Myeloid Leukemia (AML) cells

		**RT-qPCR**		**Affymetrix**		**THP-1**		**EOL-1**		**MV4-11**		**RS4-11**	
		**n = 9**		**n = 206**		**n = 3**		**n = 3**		**n = 3**		**n = 3**	
	**Name**	**% Mean**	**SEM**	**Mean**	**SEM**	**% Mean**	**SD**	**% Mean**	**SD**	**% Mean**	**SD**	**% Mean**	**SD**
A. Class Cys-Based PTPs													
A.1. Classical PTPs													
A.1.1. Transmembrane Classical PTPs													
	***PTPRA***	**8.4**	0.6	**268.5**	3.5	**3.6**	0.3	**5.9**	2.1	**4.5**	0.7	**14.2**	9.1
	*PTPRB*	n.d.		26.5	0.9	n.d.		n.d.		n.d.		1.2	0.5
	***PTPRC***	**51.2**	3.6	84.3	5.1	**6.5**	0.6	**28.6**	15.5	**4.6**	0.9	**48.3**	12.6
	*PTPRD*	0.1	0.0	23.3	1.4	n.d.		n.d.		n.d.		n.d.	
	*PTPRE*	5.7	0.4	83.8	3.8	2.2	1.0	0.5	0.1	1.9	0.2	1.5	0.6
	*PTPRF*	0.2	0.0	14.4	0.9	1.7	0.2	n.d.		n.d.		n.d.	
	*PTPRG*	n.d.		6.6	0.5	0.1	0.1	n.d.		0.5	0.0	0.1	0.0
	*PTPRH*	4.0	0.3	18.3	0.8	0.1	0.1	n.d.		0.1	0.0	0.0	0.1
	*PTPRJ*	3.1	0.2	26.4	1.7	**4.1**	0.7	1.9	0.3	1.7	0.7	1.2	0.3
	*PTPRK*	n.d.		20.7	0.7	0.1	0.0	n.d.		n.d.	0.0	0.2	0.1
	*PTPRM*	0.3	0.0	7.7	0.5	n.d.		n.d.		n.d.		n.d.	
	*PTPRN*	n.d.		56.9	1.4	n.d.		n.d.		n.d.		n.d.	
	*PTPRN2*	0.1	0.0	54.6	4.1	0.3	0.1	n.d.		0.2	0.1	0.1	0.0
	*PTPRO*	n.d.		7.2	0.4	n.d.		n.d.		n.d.		n.d.	
	*PTPRQ2*	3.3	0.2	N.D.	N.D.	n.d.		n.d.		n.d.		n.d.	
	*PTPRR*	4.8	0.3	7.4	0.4	0.2	0.1	0.5	0.4	0.9	0.5	**16.9**	3.3
	*PTPRS*	0.4	0.0	5.4	0.4	2.3	0.2	n.d.		n.d.		n.d.	
	*PTPRT*	n.d.		15.1	0.9	n.d.		n.d.		n.d.		n.d.	
	*PTPRU*	n.d.		64.8	1.5	0.2	0.1	0.1	0.0	0.1	0.0	n.d.	
	*PTPRZ1*	n.d.		8.9	0.5	n.d.		n.d.		n.d.		n.d.	
A.1.2. NRPTPs													
	*PTPN1*	0.2	0.0	188.2	3.3	n.d.		0.1	0.1	n.d.		0.2	0.1
	***PTPN2***	**16.2**	1.1	33.7	1.0	**4.3**	0.8	**7.7**	1.4	**6.1**	0.2	**10.5**	0.7
	*PTPN3*	n.d.		26.5	1.0	0.3	0.1	n.d.		n.d.		n.d.	
	*PTPN4*	2.0	0.1	145.7	5.4	0.4	0.1	0.4	0.1	0.2	0.0	1.2	0.3
	*PTPN5*	n.d.		2.8	0.1	n.d.		n.d.		n.d.		n.d.	
	***PTPN6***	**43.4**	3.0	**1357.1**	38.0	**12.6**	2.8	**21.2**	2.1	**29.7**	12.2	**19.9**	2.6
	***PTPN7***	**8.0**	0.6	88.3	2.3	**6.0**	1.2	**15.8**	3.2	**8.0**	0.3	4.2	0.7
	*PTPN9*	2.1	0.1	141.3	3.0	2.1	0.6	1.3	0.1	2.1	0.3	3.2	0.4
	***PTPN11***	**9.0**	0.6	21.1	0.6	**7.2**	0.8	**8.7**	1.1	**9.1**	0.9	**17.2**	2.8
	***PTPN12***	**7.2**	0.5	**477.8**	15.5	**5.0**	1.0	**3.1**	0.3	2.2	0.6	**4.6**	0.9
	*PTPN13*	0.1	0.0	24.5	1.2	n.d.		0.5	0.2	n.d.		0.4	0.1
	*PTPN14*	0.7	0.1	21.7	1.2	n.d.		0.1	0.1	0.6	0.2	3.8	0.4
	*PTPN18*	0.6	0.0	30.6	1.4	0.1	0.0	0.1	0.0	0.3	0.1	0.2	0.0
	*PTPN21*	n.d.		5.5	0.3	0.1	0.0	n.d.		n.d.		n.d.	
	***PTPN22***	**44.6**	3.1	**248.7**	7.4	**5.8**	1.5	**5.1**	0.9	**3.6**	1.7	4.4	1.0
	*PTPN23*	6.0	0.4	32.9	1.0	2.0	0.7	1.4	0.2	1.4	0.1	2.7	0.5
													
A.2. DSPs or VH1-like													
A.2.1. MKPs													
	***DUSP1***	**85.1**	5.9	**4620.8**	156.4	1.1	0.9	1.9	2.0	0.6	0.8	**8.0**	2.0
	***DUSP2***	**14.7**	1.0	**277.4**	32.3	**3.6**	1.2	**4.2**	2.0	**2.7**	0.7	1.8	0.3
	*DUSP4*	**6.3**	0.4	5.7	0.6	0.1	0.1	0.3	0.1	0.1	0.0	0.3	0.0
	*DUSP5*	2.4	0.2	150.4	10.1	0.2	0.1	0.3	0.2	0.4	0.2	n.d.	
	***DUSP6***	**52.2**	3.6	**1457.9**	78.0	**5.2**	1.7	**5.5**	0.4	**10.8**	3.5	2.0	0.6
	***DUSP7***	4.6	0.3	27.9	1.5	**7.4**	1.0	**5.0**	0.7	**8.0**	2.8	2.1	0.4
	*DUSP8*	**7.5**	0.5	45.5	1.7	n.d.		n.d.		n.d.		0.1	0.1
	*DUSP9*	1.1	0.1	18.3	0.7	0.2	0.1	0.1	0.0	n.d.		n.d.	
	***DUSP10***	**10.5**	0.7	**335.2**	14.2	0.6	0.4	2.8	0.4	1.2	0.5	1.3	0.3
	*DUSP16*	0.6	0.0	74.1	2.5	0.6	0.2	0.1	0.0	0.1	0.1	0.9	0.4
	***MK-ST***	5.4	0.4	131.1	3.1	**3.3**	0.4	2.0	0.1	**4.7**	0.7	**6.8**	1.9
A.2.2. Atypical DSPs													
	***DUSP3***	5.0	0.4	**502.8**	16.5	**3.8**	0.2	**5.9**	1.2	**5.1**	1.1	1.6	0.4
	*DUSP11*	3.5	0.2	**239.3**	4.7	0.7	0.1	1.4	0.2	1.1	0.1	2.7	0.1
	***DUSP12***	**6.9**	0.5	**211.2**	3.0	**4.4**	0.4	**5.7**	1.1	**4.1**	0.3	**7.3**	2.2
	*DUSP14*	5.4	0.4	114.4	3.4	1.0	0.1	2.0	0.3	1.1	0.1	2.2	0.3
	*DUSP15*	2.4	0.2	26.7	0.9	0.1	0.1	n.d.		n.d.		0.8	0.2
	*DUSP18*	1.1	0.1	9.3	0.4	0.1	0.0	0.1	0.0	0.1	0.0	0.2	0.1
	*DUSP19*	0.2	0.0	16.8	0.8	0.1	0.0	0.1	0.0	0.1	0.0	0.9	0.1
	***DUSP22***	3.1	0.2	**317.9**	6.8	0.3	0.2	1.0	0.2	1.1	0.3	**5.6**	1.0
	***DUSP23***	**6.5**	0.5	86.2	3.1	**3.5**	0.0	2.5	0.3	**2.9**	0.4	**4.7**	0.5
	*EPM2A*	4.4	0.3	89.4	2.0	0.3	0.1	0.6	0.0	0.4	0.1	1.5	0.4
	***PTPMT1***	**7.8**	0.5	128.2	3.1	**3.3**	0.3	**3.9**	0.4	**4.3**	0.3	**8.2**	0.8
	*RNGTT*	1.9	0.1	162.4	2.4	1.4	0.3	2.2	0.5	1.5	0.2	2.7	0.3
	***STYX***	5.3	0.4	**279.5**	5.3	**4.1**	0.4	**4.1**	0.8	**5.1**	0.6	**6.4**	1.2
A.2.3. Slingshots													
	*SSH1*	1.5	0.1	45.9	1.4	0.5	0.1	0.8	0.1	0.5	0.0	0.6	0.1
	***SSH2***	**7.6**	0.5	48.5	2.0	1.9	0.3	**3.0**	1.1	**2.5**	0.4	4.6	0.9
	*SSH3*	1.6	0.1	143.0	3.5	1.0	0.1	0.9	0.3	1.0	0.1	1.1	0.2
A.2.4. PRLs													
	***PTP4A1***	**18.8**	1.3	**444.3**	14.3	**17.2**	1.3	**19.4**	4.8	**8.9**	1.4	**39.8**	6.5
	***PTP4A2***	**50.2**	3.5	**1323.2**	32.7	**21.2**	2.4	**28.8**	2.7	**22.1**	3.4	**31.8**	7.3
	*PTP4A3*	1.6	0.1	92.2	7.7	0.2	0.1	1.5	0.0	1.7	0.4	0.2	0.0
A.2.5. CDC14s													
	*CDC14A*	3.1	0.2	98.2	2.9	1.3	0.2	1.0	0.3	0.9	0.1	1.2	0.3
	*CDC14B*	n.d.		25.0	1.0	0.2	0.0	n.d.		0.1	0.0	n.d.	
	*CDKN3*	1.3	0.1	103.6	6.3	1.1	0.3	2.4	0.5	1.9	0.7	3.4	1.1
	*PTP9Q22*	0.3	0.0	8.2	0.5	0.2	0.0	0.1	0.0	0.4	0.0	0.6	0.1
A.2.6. PTENs													
	***PTEN***	**12.3**	0.9	**641.8**	13.4	**5.4**	1.0	**5.4**	1.4	**9.4**	1.8	**8.5**	2.2
	*TNS1*	2.3	0.2	16.2	2.7	0.5	0.0	n.d.		0.6	0.3	0.7	0.4
	*TNS3*	2.0	0.1	**305.5**	12.6	1.1	0.2	2.7	0.9	1.1	0.1	0.5	0.1
	*TENC1*	n.d.		31.5	1.4	n.d.		n.d.		n.d.		n.d.	
A.2.7. Myotubularins													
	*MTM1*	1.6	0.1	109.4	2.1	1.0	0.2	1.2	0.5	0.8	0.1	0.6	0.1
	*MTMR1*	0.8	0.1	**316.0**	6.4	0.6	0.2	0.7	0.2	0.3	0.0	0.8	0.1
	***MTMR2***	3.1	0.2	**201.8**	5.3	**3.5**	0.2	**3.2**	0.2	**3.1**	0.1	**6.9**	0.5
	*MTMR3*	3.3	0.2	**272.5**	6.7	1.5	0.4	0.7	0.1	0.9	0.3	2.9	0.8
	***MTMR4***	2.0	0.1	**222.9**	3.8	2.9	0.5	1.8	0.1	1.3	0.2	**5.8**	0.1
	***MTMR5***	**8.6**	0.6	12.1	0.8	1.9	0.6	2.6	0.3	**2.6**	0.4	**6.0**	1.6
	***MTMR6***	2.2	0.2	**326.6**	10.1	1.5	0.1	**3.2**	0.9	1.3	0.2	2.1	0.3
	*MTMR7*	n.d.		12.0	0.5	n.d.		n.d.		n.d.		n.d.	
	*MTMR8*	0.1	0.0	20.4	0.6	0.1	0.0	0.1	0.0	0.1	0.0	n.d.	
	*MTMR9*	1.5	0.1	113.9	2.7	1.2	0.1	1.4	0.3	1.7	0.3	1.7	0.4
	*MTMR10*	2.6	0.2	14.6	1.1	1.4	0.2	1.1	0.2	1.1	0.3	**4.8**	1.1
	*MTMR11*	n.d.		88.8	10.7	n.d.		n.d.		n.d.		n.d.	
	*MTMR12*	3.9	0.3	103.4	1.8	2.0	0.2	2.4	0.4	2.4	0.3	**8.0**	1.5
	*MTMR13*	1.0	0.1	118.5	5.3	0.2	0.1	0.4	0.1	0.2	0.1	1.8	0.6
B. Class II Cys-Based PTPs													
	***ACP1***	**24.5**	1.7	18.7	0.7	**11.9**	1.1	**21.5**	1.8	**18.1**	0.9	**52.7**	2.6
C. Class III Cys-Based PTPs													
	*CDC25A*	0.9	0.1	67.3	3.4	2.6	0.2	2.9	0.3	1.8	0.7	4.3	1.0
	*CDC25B*	2.5	0.2	**285.0**	11.3	0.4	0.1	2.2	0.4	1.8	0.1	2.4	1.0
	*CDC25C*	0.4	0.0	44.4	1.8	0.4	0.1	0.8	0.1	0.9	0.1	1.0	0.3

mRNA expression of 92 members of the PTP family was assessed by RT-qPCR analysis in AML patient primary cells (duplicate determinations of the indicated sample number, means in % of 3 housekeeping genes ± SEM). For comparison, PTP mRNA expression data for AML patient blasts were retrieved from an Affymetrix analysis (means ± SEM). PTP gene expression was also analyzed by RT-qPCR in four AML cell lines (3 experiments with duplicate determinations, means in % of 3 housekeeping genes ± SD). The 25% highest values in the respective sample type are highlighted in bold. n.d. – not detectable. Names of PTPs, which fall in this category in at least two different sample types, or only in one sample type, are labeled bold or by underlining, respectively. Classification was taken from Alonso et al. [21].

### Role of FLT3 ITD for PTP expression

**Figure 1:**
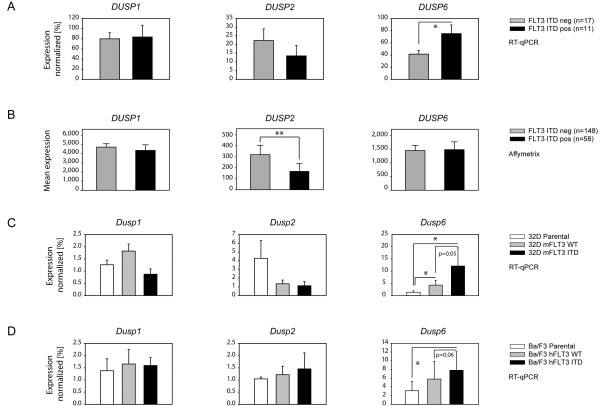
**DUSP6 mRNA levels are elevated in cells with FLT3 ITD.** mRNA expression of *DUSP1*, *DUSP2*, and *DUSP6* in primary AML cells was analyzed by RT-qPCR (**A**) or retrieved from Affymetrix data (**B**). mRNA expression of the murine genes was also analyzed by RT-qPCR in 32D cells (**C**) and BA/F3 cells (**D**), either parental or expressing wildtype (WT) FLT3 or FLT3 ITD, as indicated. RT-qPCR data are based on duplicate determinations for the indicated sample numbers (A), and on duplicate or triplicate determinations in 6 (C) or 8 (D) independent experiments, and are presented as percentage of expression of the mean of 3 housekeeping genes. Values are expressed as means + SEM (A), means + 2SEM (B), or means + SD (C, D). Statistic significance was tested by Mann–Whitney test (A, B) or Students’ test (C, D) using the software SPSS (*p < 0.05, **p < 0.01). The different relative expression levels depicted in (A) versus panels (C, D) presumably relate to the different species background (human in 1A, murine in 1 C, D), including different expression levels of housekeeping genes used for reference, and use of primary patient cells (1A) versus established cell lines (1 C, D). *DUSP* expression levels were commonly lower in cell lines compared to primary cells (see Table 1).

Specific genetic lesions in the AML cells could have an impact on PTP gene expression. We were particularly interested in a possible effect of FLT3 ITD. When mRNA expression was compared using the initially analyzed set of 9 patients (five FLT3 wildtype, four FLT3 ITD), some PTPs appeared downregulated, including the abundantly expressed DUSP species *DUSP1* and *2* (data not shown). Conversely, *DUSP6* appeared elevated in expression. These DUSPs were therefore subjected to RT-qPCR analysis for a larger number of patient samples to compare patients with wildtype FLT3 (n = 17), with patients harboring FLT3 ITD (n = 11). Also, patients positive or negative for the FLT3 ITD mutation of the Affymetrix data set were compared for the expression of these DUSPs. As shown in Figure 1 A, B, downregulation of *DUSP2* expression was seen as a trend in the RT-qPCR analysis and was significant in the Affymetrix data set. Upregulation of *DUSP6* with FLT3 ITD could not be seen in the Affymetrix set, but was significant in the RT-qPCR analysis. The initially observed apparent alterations in *DUSP1* expression could not be confirmed with larger sample numbers. To test if the changes in DUSP expression were indeed caused by the presence of FLT3 ITD, we included also 32D and Ba/F3 cell lines, parental or stably expressing wildtype FLT3, or FLT3 ITD. As shown in Figure 1 C, D, alterations of *DUSP1* mRNA levels in dependence of FLT3 ITD expression were not observed. Likewise, regulation of *DUSP2* expression could not be correlated with presence of FLT3 ITD in these cell lines, whereas induction of *DUSP6* mRNA in presence of FLT3 ITD was clearly seen in both cell backgrounds.

### DUSP6 expression is maintained by FLT3 ITD signaling activity

**Figure 2:**
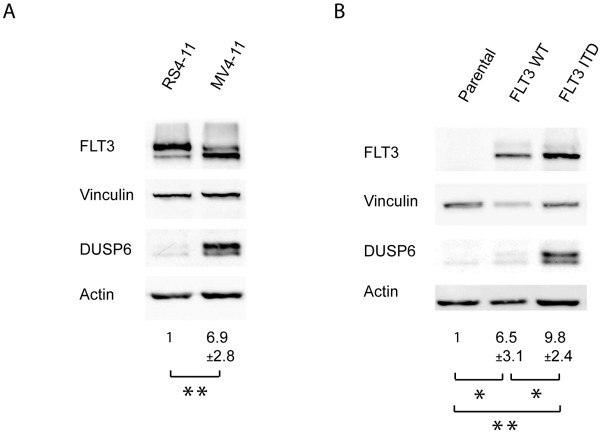
**FLT3 ITD expressing cells exhibit high DUSP6 protein expression.** Lysates of different cell lines (as indicated) were subjected to SDS-PAGE and immunoblotting to evaluate DUSP6 protein levels. For loading control, levels of vinculin and β-actin were also detected, and FLT3 levels were assessed for comparison. (**A**) RS4-11 cells, endogenously expressing wildtype FLT3, were compared with MV4-11 cells, endogenously expressing FLT3 ITD. Numbers under the lanes represent mean values (± SD) for the quantification of blots of 3 separate experiments (RS4-11 set to 1.0). (**B**) 32D cell lines either mock-transfected (“parental”), or stably transfected with wildtype FLT3 (WT) or FLT3 ITD (as indicated) were compared. Numbers represent quantification of 4 separate experiments (DUSP6 signal normalized to actin or vinculin; mean ± SD, parental set to 1.0; statistic significance was tested by Students’ test (*p < 0.05, **p < 0.01).

**Figure 3:**
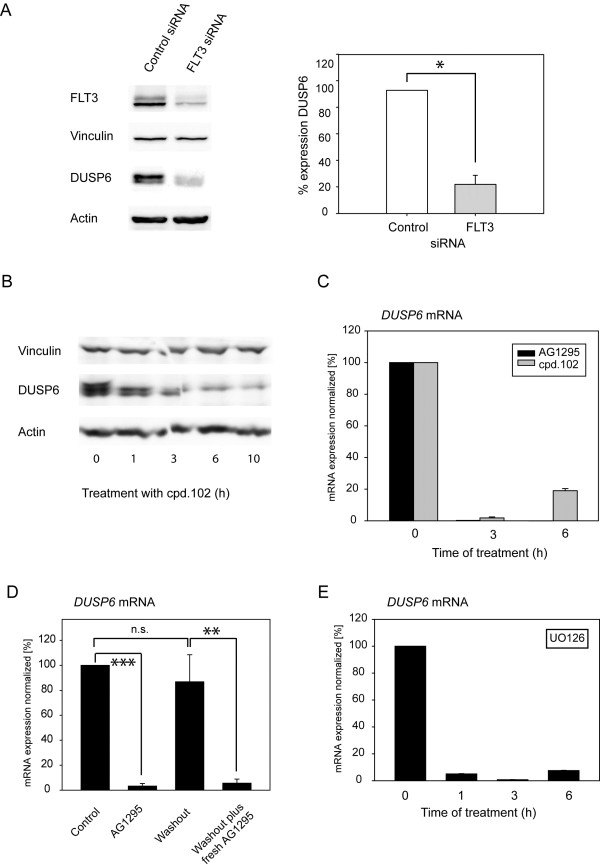
**FLT3 ITD signaling drives DUSP6 expression.** MV4-11 cells endogenously expressing FLT3 ITD were subjected to different treatments. (**A**) Cells were transiently transfected with FLT3-specific siRNA using “Smart pool” siRNA and the nucleofection technology. After 48 h, DUSP6 protein levels were assessed by immunoblotting. Left panel: example experiment. Right panel: quantification of 2 independent experiments (*p < 0.05 by t-test). (**B**) MV4-11 cells were treated with the FLT3 inhibitor cpd.102 (1 μM) in complete medium for the indicated time periods. DUSP6 levels were assessed by immunoblotting. The experiment is representative of 2 with consistent results. (**C**) Cells were treated with the FLT3 inhibitors cpd.102 (1 μM), or AG1295 (20 μM), as indicated, in complete medium for different time periods. After treatment, cells were harvested, RNA was prepared, and *DUSP6* expression was assessed by RT-qPCR relative to *ACTB* mRNA. Values are means + SD of duplicate determinations and are representative of 3 experiments with consistent results. (**D**) To assess reversibility of the inhibition of *DUSP6* mRNA expression, cells were treated for 4 hours with AG1295 or solvent as in (C). Aliquots of the cells were directly subjected to analysis of *DUSP6* mRNA expression (as indicated). The AG1295-treated cells were washed, and either cultivated without inhibitor (washout) or with freshly added inhibitor (as indicated) for another 4 h before analysis of *DUSP6* mRNA expression. Values are means + SD of 4 independent determinations performed in triplicate. Statistic significance was tested by Students’ test (**p < 0.01, ***p < 0.001). (**E**) Cell treatments were performed with the MEK inhibitor UO126 (10 μM), and analysis carried out as in (C).

To further analyze the link of *DUSP6* expression and FLT3 ITD signaling, we employed MV4-11 cells, endogenously expressing FLT3 ITD, RS4-11 cells endogenously expressing wildtype FLT3, and 32D cells without or with expression of wildtype FLT3 or FLT3 ITD. Consistent with the mRNA data (Table 1, Figure 1C), MV4-11 cells contained clearly higher levels of DUSP6 protein than RS4-11 cells (Figure 2A). Similarly, 32D cells with FLT3 ITD had the highest DUSP6 protein expression, however, wildtype FLT3 expressing cells also showed increased DUSP6 protein levels when compared to parental 32D cells, possibly caused by basal activity of the overexpressed FLT3 (Figure 2B). To further explore a causal relationship between FLT3 ITD expression and activity and high DUSP6 expression, MV4-11 cells were employed. When endogenous FLT3 ITD in MV4-11 cells was knocked down by siRNA, the DUSP6 level was strongly reduced (Figure 3A). Moreover, inhibition of FLT3 ITD kinase activity with the selective tyrosine kinase inhibitor cpd.102 reduced DUSP6 protein as well as *DUSP6* mRNA levels in MV4-11 cells drastically (Figure 3B, C). Similarly, AG1295, another FLT3 kinase inhibitor, blocked *DUSP6* mRNA expression in FLT3 ITD expressing cells. Downregulation of DUSP6 mRNA by AG1295 was reversible, and recovered to control levels 4 hours after inhibitor washout (Figure 3D). It has previously been shown that DUSP6 expression downstream of different stimuli [22-24] is activated by ERK1/2 signaling. We therefore also tested the role of this pathway in the maintenance of high DUSP6 levels in cells with FLT3 ITD. Treatment of MV4-11 cells with the MEK inhibitor UO126, which abrogates ERK1/2 activation, indeed suppressed *DUSP6* mRNA expression almost completely (Figure 3E). Similar observations were made in 32D cells expressing FLT3 ITD (not shown). Taken together, we conclude that FLT3 ITD signaling activity causes high DUSP6 levels in AML cells, mediated by its kinase activity and downstream signaling via the ERK pathway.

### DUSP6 modulates FLT3 ITD dependent ERK1/2 signaling and cell proliferation

**Figure 4:**
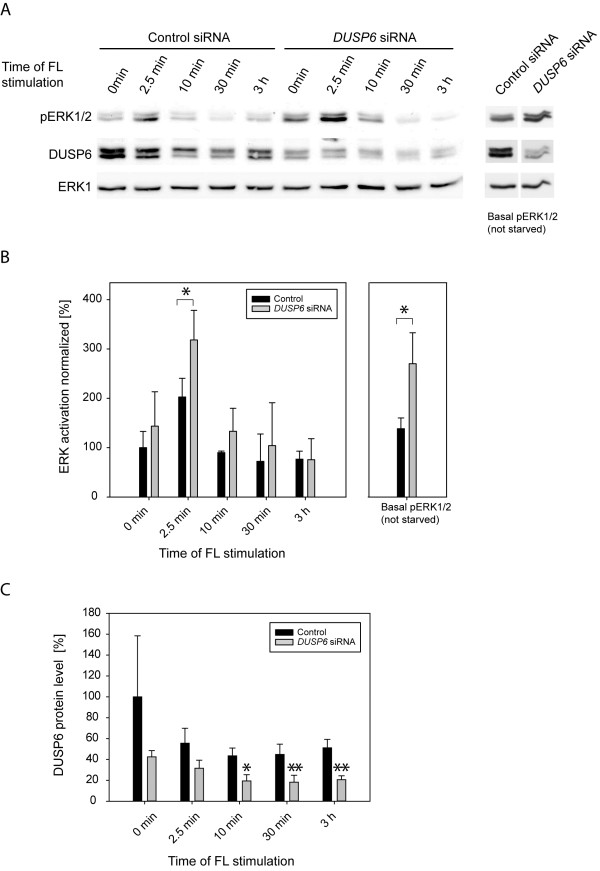
**ERK1/2 activation by FLT3 ITD in MV4-11 cells is negatively regulated by DUSP6.** MV4-11 cells were transiently transfected with *DUSP6*-targeting siRNA by nucleofection. After 48 h, cells were serum-starved for 4 h and then stimulated with 100 ng/ml FL for the indicated times. Cells were extracted and subjected to immunoblotting for detection of ERK1/2 phosphorylation, and DUSP6 levels. ERK1 was assessed as loading control. (**A**) Representative experiment. Unstimulated cells, which were cultivated in complete medium, were also analyzed (as indicated). The data for the latter are from the same blot with identical exposure and image processing, but were rearranged for better clarity. (**B**) Quantification of immunoblots for 4 independent experiments (except time-point 10 min, n = 2). Statistic siginificance was tested by Students’ test (*p < 0.05) for *DUSP6* siRNA versus control. (**C**) Effect of FL stimulation on DUSP6 protein levels. Quantification of immunoblots for 4 independent experiments. DUSP6 signals were normalized to ERK1. Statistic significance was tested by Students’ test (*p < 0.05,**p < 0.01) for difference from unstimulated control.

**Figure 5:**
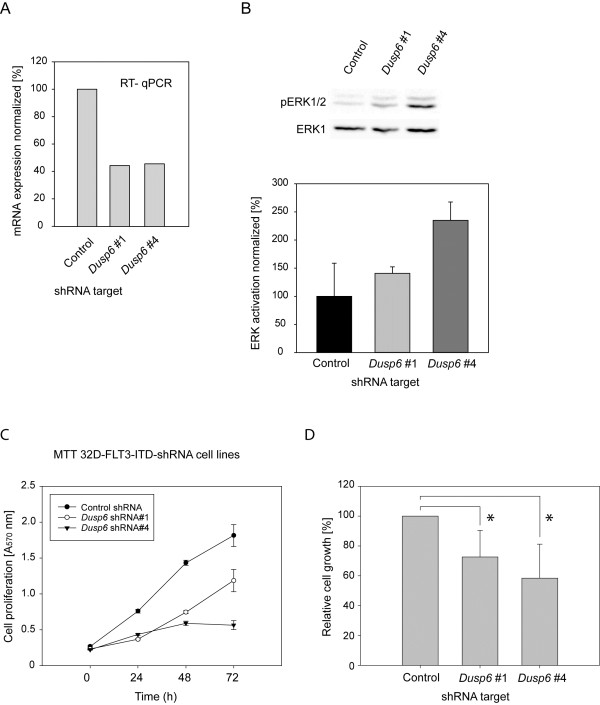
**DUSP6 attenuates ERK1/2 activity but positively regulates proliferation of FLT3 ITD expressing 32D cells.** 32D cells stably expressing FLT3 ITD were subjected to transduction with lentiviral particles encoding shRNA targeting *Dusp6*. Transduced cell pools were selected, and levels were assessed by RT-qPCR for *Dusp6* mRNA (relative to *Actb*) (**A**). The effect of *Dusp6* knockdown for the indicated shRNAs on ERK1/2 phosphorylation was assessed in serum-starved cells by immunoblotting (**B**). Upper panel: Representative experiment, lower panel: quantification of 2 experiments (means + SD). pERK1/2 signals were normalized to ERK1 signals. Cell pools transduced with *Dusp6*-targeting shRNA were subjected to proliferation assays in 0.5% FCS in absence of cytokines with the MTT method. (**C**) Representative experiment, values are means of 7 replicas ± SD. (**D**) Summary of 4 independent experiments. Values at 72 h obtained for the two different *Dusp6* shRNAs were normalized to the amounts measured in the individual experiments for the control shRNA cell pool (100%) (means + SD, n = 4, Statistic significance was tested by Students’ test (*p < 0.05).

To explore the role of DUSP6 for FLT3 ITD mediated signaling and cell transformation, we chose to deplete MV4-11 cells of DUSP6 by using siRNA. As shown in Figure 4A, DUSP6 protein levels could be greatly reduced, but not completely abolished in MV4-11 cells by transient siRNA transfection. ERK1/2 activity under basal growth conditions was clearly elevated. ERK1/2 activation was also analyzed upon FL stimulation. Selected time-points were chosen based on preliminary kinetic experiments showing maximum stimulation at 2.5-5 min, followed by decrease of the signal and partial recovery after prolonged stimulation of 1–6 h (not shown). ERK1/2 phosphorylation under these conditions was also elevated by DUSP6 knockdown, albeit moderately reaching significance only at 2.5 min (Figure 4A,B). Interestingly, FL stimulation was associated with a decrease in the DUSP6 protein levels, which occurred relatively rapidly and was noticeable both in presence of control siRNA as well as in cells with downregulated DUSP6 levels (Figure 4A, C). To enable assessment of biological readouts, we also performed stable transduction of FLT3 ITD expressing 32D cells with *Dusp6*-specific shRNA. Five different target sequences of the MISSION® shRNA collection were tested: Two of them (*Dusp6* targets #1 and #4) reduced *Dusp6* mRNA expression to 40-50% (Figure 5A). The relative moderate success of *Dusp6* knockdown under these condidions is presumably related to feedback regulation: enhanced ERK1/2 signaling upon *Dusp6* knockdown is expected to stimulate *Dusp6* expression. We characterized cell pools transduced with these shRNAs further. As expected, ERK1/2 activation in these cells was increased (Figure 5B). Interestingly, the cell pools with attenuated *Dusp6* expression grew significantly slower (Figure 5C,D), despite the moderately elevated ERK1/2 activity. Notably, growth was assessed in absence of cytokines, therefore depending completely on FLT3 ITD signaling. Thus, high DUSP6 levels in FLT3 ITD expressing cells attenuate ERK1/2 activation, however, do not inhibit but rather appear to promote cell proliferation.

## Discussion

By RT-qPCR analysis in primary AML cells, AML cell lines, and evaluation of Affymetrix data we have established a profile of PTP expression in AML. Highly expressed genes include three transmembrane classical PTPs and 6 non-receptor classical PTPs. Interestingly, several dual-specificity PTPs (DUSPs) were found abundantly expressed, suggesting important functions. This applies also to *PTP4A1* and *PTP4A2* (PRL-1 and −2), and *ACP1* (LMW-PTP). The possible roles of some of these PTPs for cell biology in leukemia have been previously reviewed [25] but further functional investigation of these highly expressed PTPs appears warranted. PTP mRNA expression in different types of immune cells has been recently evaluated [26]. This study has defined a set of PTPs commonly expressed in immune cell lineages. As one could expect, this set shows many overlaps with the PTP profile in AML cells described here, for example *PTPRC*/CD45 and *PTPN6*/SHP-1 are among the highly expressed genes in both analyses. However, certain of these “commonly expressed” PTP genes in immune cells were only weakly or not detected in our analysis, including for example *PTPRFPTPR*S, *PTPN3PTPN9*, or *DUSP4*. These PTPs may not be expressed in the early differentiation stages represented by the leukemic blasts, or may potentially be downregulated, another interesting topic for further investigation. Some obvious discrepancies in the expression levels of PTP genes in the patient samples either detected by RT-qPCR or by Affymetrix analysis were apparent. They may mostly relate to the different detection of splice versions by the two methods, and in part to the different sample sets. Still, of the 23 PTP genes detected among the upper 25% by expression level, 12 match for both detection methods. For the case of DUSP6, RT-qPCR analysis was clearly advantageous over the microarray technique to detect relevant and further validated changes.

We have focused our interest on the possible role of FLT3 ITD, a common oncogenic lesion in AML, on PTP expression. It appeared possible that changed PTP expression would contribute to FLT3 ITD mediated transformation. Only relatively few alterations of PTP expression were, however, observed. Notably, *PTPN6* (SHP-1), a highly expressed PTP with a proven negative regulatory role for cytokine signaling and possible role in regulation of FLT3 was not altered in mRNA expression in our data sets, contrary to what has previously been proposed [17]. Also, *PTPRJ*, a negative regulator of FLT3 autophosphorylation [11], was not altered in expression by FLT3 ITD (data not shown). As we have found recently, FLT3 ITD appears, however, to inactivate PTPRJ by production of high levels of reactive oxygen species [27]. *DUSP2* mRNA was downregulated in primary AML cells with FLT3 ITD, but this phenomenon could not be recapitulated in 32D or Ba/F3 cells. It is possible that further lesions in the primary AML cells contribute to *DUSP2* regulation. Still, reduced DUSP2 levels may play a role in FLT3 ITD-transformed cells, which we have not yet explored.

Interestingly, *DUSP6* was elevated in expression downstream of FLT3 ITD signaling, as found in our RT-qPCR analysis of primary AML cells, as well as in different model cell systems. DUSP6 induction at mRNA and protein level could be causally linked to FLT3 ITD signaling activity and ERK1/2 pathway activation. Upregulation of *DUSP6* mRNA was, however, not detectable in the Affymetrix data set. It is possible that this method is not sufficiently sensitive to detect the FLT3 ITD-mediated alteration in *DUSP6* expression. In patients with activating N-RAS mutations, an increase in *DUSP6* expression could, however, be seen in the Affymetrix data set (not shown), possibly indicating that N-RAS mutations are more potent than FLT3 ITD in driving *DUSP6* expression. Previous gene expression analyzes (also using Affymetrix expression arrays) in myeloma cells have identified *DUSP6* as one of only three genes which were uniquely and strongly elevated in cells harboring activated N-RAS [28]. Interestingly, other stimuli which only transiently activated ERK signaling such as interleukin-6 stimulation did not induce a sufficiently robust *DUSP6* response to allow detection with this technique. Consistent with our findings, *DUSP6* has previously been identified as one of the genes which are most effectively downregulated in FLT3 ITD expressing AML cells treated with the FLT3/broad spectrum kinase inhibitor CEP701 (Lestaurtinib) [29]. Based on its capacity for dephosphorylating the pThr-X-pTyr motif in ERK1/2 [19], DUSP6 can negatively regulate pERK1/2 levels in multiple cell types. Since the ERK1/2 pathway mediates mitogenic signaling, among other responses, DUSP6 has been proposed as a tumor suppressor [23]. Notably, in pancreatic cancer, DUSP6 levels are downregulated by gene deletion or promoter hypermethylation, consistent with such a function [30-32]. Recently, DUSP6 was shown to inhibit growth, migration and epithelial-to-mesenchymal transition (EMT) of esophagal squamous cell carcinoma and nasopharyngeal carcinoma cells [33]. In non-small cell lung cancer, however, high DUSP6 levels have been found to predict poor prognosis, and evidence for a tumor-promoting role of DUSP6 in human glioblastoma has recently been provided [34,35]. Moreover, DUSP6 has recently been found as a predictor of invasiveness in papillary thyroid cancer. Silencing of DUSP6 expression decreased the cell viability and migration rate of a corresponding cell line [36]. As shown in the present study, elevated DUSP6 levels correlate with the presence of FLT3 ITD, a negative predictor of prognosis [5] in AML cells. This observation, taken together with the functional findings discussed below, indicates that DUSP6 may play a pro-oncogenic role in FLT3 ITD-positive AML. Obviouosly, depending on the context, DUSP6 affects tumor biology in very different ways.

The function of high DUSP6 levels in FLT3 ITD expressing cells was addressed by RNAi experiments. Consistent with its function as ERK1/2-PTP, downregulation of DUSP6 caused elevated constitutive ERK1/2 activation in FLT3 ITD expressing cells. Moreover, ERK1/2 phosphorylation was also elevated in FL-stimulated cells, albeit only moderately. We observed that FL-stimulation caused downregulation of DUSP6 protein levels, similar as it has been described earlier for the stimulation of cells with other mitogens. In these studies the authors linked the reduction of DUSP6 levels to proteasomal degradation, which was prompted by phosphorylation at serine residues [22,37]. Surprisingly, 32D cell pools with attenuated *Dusp6* expression were, however, moderately impaired in proliferation, indicating that high DUSP6 levels may not play a negative but rather some positive role in FLT3 ITD dependent cell growth. Forced overexpression of exogenous DUSP6 in stable transfectants reduced ERK1/2 phosphorylation, but did not inhibit cell proliferation (data not shown), further supporting the notion that ERK1/2 activity and cell growth in FLT3 ITD-expressing cells are not simply positively correlated. It is well known that the biological outcome of ERK1/2 activation depends both on its magnitude and on its kinetics, which are determined by several feedback mechanisms [38]. For example, sustained high ERK1/2 activation by the phorbol ester TPA in MCF7 cells causes growth arrest [24]. Similarly, nerve growth factor (NGF) stimulation of PC-12 pheochromocytoma cells as opposed to epidermal growth factor (EGF) stimulation leads to sustained high-level ERK1/2 activation and not a proliferative, but a differentiation response [39,40]. The alternate cellular responses to different kinetics and magnitude of ERK1/2 activation seem based on differential transcriptional sensing [41], which is likely to depend on the specific cell type and the simultaneous activation of other signaling pathways. In the context of FLT3 ITD, high constitutive expression of DUSP6 appears associated with a relatively low but constitutive level of ERK1/2 activity, which is compatible with efficient cell growth. ShRNA-mediated DUSP6 downregulation causes a higher level of constitutive ERK1/2 activation (see Figure 4), which, similar as in the case of MCF7 or PC12 cells, may ultimately result in diminished cell proliferation by yet unclear downstream mechanisms. Alternatively or in addition, potential other substrates of DUSP6 may play a role. These possibilities need to be further explored.

## Conclusions

Elevated expression of the dual-specificity phosphatase DUSP6 is driven by the oncoprotein FLT3 ITD in AML. Despite the known role of DUSP6 as negative regulator of ERK1/2 activation, which was also observed in FLT3 ITD-expressing leukemia cells, DUSP6 was identified as a positive component in the FLT3 ITD-driven proliferation. This finding prompts its further characterization as a possible drug target.

## Methods

### Cell lines

The human AML cell lines MV4-11, THP1, EOL1, RS4-11 were obtained from the German Collection of Microorganisms and Cell Cultures (DSMZ, Braunschweig, Germany) and maintained in RPMI 1640 medium with glutamine (PAA, Cölbe, Germany) supplemented with 10% heat inactivated fetal calf serum (FCS, BioWest, Berlin, Germany). Murine 32D cells stably expressing exogenous wild type mFLT3 or mFLT3 ITD were kindly provided by Drs. Justus Duyster and Rebekka Grundler (Klinikum Rechts der Isar, Munich, Germany), and were routinely cultured in RPMI 1640 medium with HEPES (Biochrome Berlin, Germany) supplemented with 10% heat inactivated FCS, 1 mM sodium pyruvate, and 2.5 ng/ml murine recombinant IL3. Murine Ba/F3 cells stably expressing exogenous wildtype hFLT3 or hFLT3 ITD were kindly provided by Dr. Lars Rönnstrand (Experimental Clinical Chemistry, University Hospital Lund/Malmö, Sweden) and were cultured under the same conditions as 32D cells. To assess *Dusp6* mRNA or DUSP6 protein expression (experiments shown in Figures 1C,D, and 2B, respectively) or FLT3 ITD-dependent growth (e.g. experiments shown in Figure 5C, D), cultivation was done in absence of cytokines.

### Antibodies and reagents

The polyclonal DUSP6 antibody used in this study has been described earlier [22]. Mouse monoclonal anti-β-actin antibody (clone AC15) was from Sigma Aldrich (A1978, Deisenhofen, Germany), mouse monoclonal anti-ERK1 antibody was from Transduction Laboratories (M12320/L1), mouse monoclonal anti-phospho-ERK1/2 antibody from Cell Signaling (Frankfurt, Germany), FLT3 Polyclonal anti-FLT3 antibody (C-20, sc-479) was from Santa Cruz Biotechnology (Heidelberg, Germany) and mouse monoclonal anti-Vinculin (BZL03106) antibody was from Biozol Diagnostic (Eching, Germany). Horseradish peroxidase-conjugated secondary antibodies were from KPL (Wedel, Germany). AG1295 was purchased from Alexis Biochemicals (Grünberg, Germany). FLT3 ligand (FL, human), and murine IL-3 were from Peprotech (London, UK). The MEK inhibitor UO126 was from Tocris Bioscience (Bristol, UK). The bisindolylmethanone FLT3 inhibitor cpd. 102 has been described earlier [42].

### Cell treatments and preparation of RNA and protein samples

To evaluate basal PTP expression in human AML cell lines, 1–2 x 10^6^ cells were harvested from well-growing cultures by centrifugation at 300 x g for 5 minutes. The supernatant was discarded and the cell pellets were used for total RNA preparation using the RNeasy kit (Qiagen, Hilden, Germany). Biological replicas were prepared with independently cultured cell batches. 32D cells and Ba/F3 cells were starved from cytokines by washing them twice and subsequently incubating them with RPMI medium containing 0.5% FCS for 4 h before isolating RNA. RS4-11 and MV4-11 cells were starved with serum-free RPMI medium for 4 h before FL stimulation. For protein extraction, cells were centrifuged (300 x g, 5 min). The pellets were washed once with ice cold PBS, and then extracted with 60–80 μl lysis buffer. containing 50 mM HEPES (pH 7.4), 150 mM NaCl, 1 mM EDTA, 25 mM NaF, 1% (v/v) NP-40 and (freshly added) aprotinin, 65 KIU/ml, 1 μg/ml leupeptin, 1 μg/ml pepstatin, 1 mM PMSF, 0.2 mg/ml Pefabloc, and 1 mM sodium orthovanadate.

### Human AML samples

AML patients included in this study were diagnosed and treated either at the Jena University Hospital (Jena, Germany) or at the Otto-von-Guericke University Magdeburg (Magdeburg, Germany). The retrospective molecular analyses of leukemic blasts were approved by the institutional review boards of each university hospital. AML cells were isolated from peripheral blood or bone marrow from AML patients at diagnosis after informed consent. In part, bone marrow was frozen in FCS supplemented with 10% IMDM and 10% DMSO until workup. Erythrocytes were lysed using erythrocyte lysis buffer (QIAGEN, Hilden, Germany) according to the manufacturer's instructions, and blasts were purified by Ficoll (Biochrom, Berlin, Germany) density gradient separation. RNA isolation was carried out with the RNeasy Mini Kit (QIAGEN, Hilden, Germany). FLT3 mutation status was determined by RT-PCR using standard diagnostic primers ITD1 (5´- GCAATTTAGGTATG AAAGCCAGC-3105294) and ITD2 (5´- CTTTCAGCATTTTGACGGCAACC-3´) or FLT3-ITD-fw 5-GCAATTTAGGTATGAAAGCCAGC-3 and FLT3-ITD-rev 5-CTTTCAGCATT TTGACGGCAACC-3.

### Expression analysis by RT-qPCR

The platform for PTP mRNA expression analysis by RT-qPCR has been described recently [18]. RNA was quantified by UV measurement, and integrity was verified using an Agilent 2100 Bioanalyzer. Samples with concentrations < 100 ng/μl, A_260_:A_280_ ratios <2.0, or partially degraded RNA were excluded from further processing. 1 μg RNA was used directly for preparation of cDNA using a BioRad iScript cDNA synthesis kit (BioRad, Munich, Germany) (for human AML samples and cell lines) or a Fermentas cDNA synthesis kit (Fermentas, St. Leon-Rot, Germany) (for all other samples) according to the manufacturer’s instructions. cDNA corresponding to 40 ng RNA input was used for the qPCR. The primers for amplification of most of the transcripts were purchased from Qiagen (QuantiTect® Primer Assay, Qiagen, Hilden, Germany), except primer sets for *huDUSP2huDUSP4huSBF1huPTPN2huPTPN22*, and *huPTPRR*, which were bought from SABiosciences (Hilden, Germany). The real-time PCR reactions were carried out using the FastStart Universal SYBR Green Master (ROX) from Roche (Mannheim, Germany) and an Applied Biosystems 7900HT Fast Real-Time PCR system (Merck Serono facility, Geneva, Switzerland), or the RTPCR, Maxima™ SYBR green from Fermentas (St. Leon-Rot, Germany, Cat. No. K0221) and an Eppendorf Realplex® Mastercycler. The conditions for PCR included 95°C for 15 min (to activate the hotstart *Taq* polymerase), followed by 40 cycles of 94°C for 15 sec, 55°C for 30 sec, 72°C for 30 sec. Threshold Cycles (Ct) were determined after the completion of PCR and calculations of the relative expression of PTP genes were done based on ΔCt values and the mean Ct of the three housekeeping genes proteasome subunit beta type-3 (*PSMB3/Psmb3*), calnexin (*CANX/Canx*), and β-actin (*ACTB/Actb*). For analyses of drug effects of *DUSP6/Dusp6* expression in cell lines, ΔCt were calculated relative to β-actin (ACTB/*Actb*) expression.

### Analysis of array data

A published microarray dataset (GSE1159) was analyzed for PTP expression levels in FLT3 WT and FLT3 ITD samples [43]. Data were analyzed as described previously [44].

### siRNA and shRNA transfections

For siRNA-mediated knockdown of human FLT3 the ON-TARGETplus SMARTpool from Dharmacon (ThermoFisher Scientific, Schwerte, Germany, Cat. No. L-003137-00-0005) was used. As control, the ON-TARGETplus non-targeting pool (Cat. No. D-001810-10-20) was used. MV4-11 cells were transfected using the Lonza (Cologne, Germany) Cell Line Nucleofector Kit V according to the instructions of the manufacturer. In brief, 2 x 10^6^ cells were taken up in 100 μl of transfection solution, 2 μg siRNA were added, and the suspension was transferred to an electroporation cuvette and pulsed once using the program A30 of the AMAXA Nucleofector. Thereafter, cells were diluted in culture medium and maintained for 48 hours before further analysis. For shRNA-mediated knockdown of DUSP6 in 32D cells, pLKO.1-based constructs were obtained from the Sigma-Aldrich (Deisenhofen, Germany) MISSION® shRNA collection: Dusp6 #1 TRC ID number TRCN0000055038 (5’CCGGCGATGCTTACGACATTGTTAACTCGAGTTAACAATGTCGTAAGCATCGTTTTTG 3’); Dusp6 #4 TRCN0000055041 5’CCGGCCTGAGGCCATTTCTTTCATACTCGAGTATGAAAGAAATGGCCTCAGGTTTTTG3’. Production of lentiviral particles and transduction and selection of 32D cell pools was done as described before [11].

### DUSP6 overexpression

A construct encoding ratDUSP6 has been described earlier [45]. It was subcloned into the vector LeGO-iC ([46], kindly provided by Dr. C. Stocking, Heinrich Pette Institute, Hamburg, Germany) by standard techniques. Stable transfection of 32D cell pools was performed as described previously [47].

### Other assays

Immunoblotting and assessment of cell proliferation using 3-(4,5-dimethylthiazol-2-yl)-2,5-diphenyltetrazolium bromide (MTT) were performed as described earlier [47]. Immunoblots were developed using enhanced chemiluminiscence and detection with a LAS4000 Imager (Fujifilm Europe GmbH, Düsseldorf, Germany). Signals were quantified using Multi Gauge V3.0 Software (Fujifilm Europe GmbH, Düsseldorf, Germany).

## Misc

Deepika Arora and Susanne Köthe contributed equally to this work.

## Competing interests

The authors declare that they have no competing interests.

## Authors’ contributions

DA, SK, MvdE and RHvH performed the PTP expression analysis and analyzed the data, FH, TF, and SS provided and worked up clinical samples, BT, and SAB performed functional studies, JL developed a critical reagent and supported the study with experimental protocols and advice, FI and CMT performed bioinformatic analysis of expression data, FDB planned and supervised the study and wrote the manuscript. All authors read and approved the final manuscript.
